# Supplementary material for: Sorting at embryonic boundaries requires high heterotypic interfacial tension
Source: Nat Commun. 2017 Jul 31;8:157. doi: 10.1038/s41467-017-00146-x (PMC5537356; doi:10.1038/s41467-017-00146-x)
Supplement: Supplementary file 2 — Supplementary Software 1 [file 41467_2017_146_MOESM2_ESM.zip › PottsModel/SrcPottsModel/doc/mvc/package-use.html]

Uses of Package mvc


JavaScript is disabled on your browser.


Skip navigation links


- Overview
- Package
- Class
- Use
- Tree
- Deprecated
- Index
- Help

- Prev
- Next

- Frames
- No Frames

- All Classes

# Uses of Package mvc

- Packages that use mvc

  | Package | Description |
  | --- | --- |
  |  |  |
  | --- | --- |
  | engine |  |
  | gui |  |
  | model |  |
  | mvc |  |
- Classes in mvc used by engine

  Class and Description

  AObservable

  Implements a class which can be extended to provide observer support.

  IObservable

  Observer

  Interface that can be implemented to provide observer support.
- Classes in mvc used by gui

  Class and Description

  Observer

  Interface that can be implemented to provide observer support.
- Classes in mvc used by model

  Class and Description

  AObservable

  Implements a class which can be extended to provide observer support.

  IObservable

  Observer

  Interface that can be implemented to provide observer support.
- Classes in mvc used by mvc

  Class and Description

  IObservable

  Observer

  Interface that can be implemented to provide observer support.

Skip navigation links


- Overview
- Package
- Class
- Use
- Tree
- Deprecated
- Index
- Help

- Prev
- Next

- Frames
- No Frames

- All Classes
